# Caffeic Acid, a Phenol Found in White Wine, Modulates Endothelial Nitric Oxide Production and Protects from Oxidative Stress-Associated Endothelial Cell Injury

**DOI:** 10.1371/journal.pone.0117530

**Published:** 2015-04-08

**Authors:** Massimiliano Migliori, Vincenzo Cantaluppi, Claudio Mannari, Alberto A. E. Bertelli, Davide Medica, Alessandro Domenico Quercia, Victor Navarro, Alessia Scatena, Luca Giovannini, Luigi Biancone, Vincenzo Panichi

**Affiliations:** 1 Nephrology and Dialysis Unit, Versilia Hospital, Lido di Camaiore, Italy; 2 Nephrology, Dialysis and Kidney Transplantation Unit, Department of Medical Sciences, University of Torino, Torino, Italy; 3 Department of Translational Research and New Technology in Medicine, University of Pisa, Pisa, Italy; 4 Department of Biomedical Sciences for Health, University of Milan, Milan, Italy; University of Missouri, UNITED STATES

## Abstract

**Introduction:**

Several studies demonstrated that endothelium dependent vasodilatation is impaired in cardiovascular and chronic kidney diseases because of oxidant stress-induced nitric oxide availability reduction. The Mediterranean diet, which is characterized by food containing phenols, was correlated with a reduced incidence of cardiovascular diseases and delayed progression toward end stage chronic renal failure. Previous studies demonstrated that both red and white wine exert cardioprotective effects. In particular, wine contains Caffeic acid (CAF), an active component with known antioxidant activities.

**Aim of the study:**

The aim of the present study was to investigate the protective effect of low doses of CAF on oxidative stress-induced endothelial injury.

**Results:**

CAF increased basal as well as acetylcholine—induced NO release by a mechanism independent from eNOS expression and phosphorylation. In addition, low doses of CAF (100 nM and 1 μM) increased proliferation and angiogenesis and inhibited leukocyte adhesion and endothelial cell apoptosis induced by hypoxia or by the uremic toxins ADMA, p-cresyl sulfate and indoxyl sulfate. The biological effects exerted by CAF on endothelial cells may be at least in part ascribed to modulation of NO release and by decreased ROS production. In an experimental model of kidney ischemia-reperfusion injury in mice, CAF significantly decreased tubular cell apoptosis, intraluminal cast deposition and leukocyte infiltration.

**Conclusion:**

The results of the present study suggest that CAF, at very low dosages similar to those observed after moderate white wine consumption, may exert a protective effect on endothelial cell function by modulating NO release independently from eNOS expression and phosphorylation. CAF-induced NO modulation may limit cardiovascular and kidney disease progression associated with oxidative stress-mediated endothelial injury.

## Introduction

Nitric oxide (NO) is a potent vasodilator, involved in the regulation of vascular homeostasis. NO inhibits platelet aggregation and adhesion, leukocyte—endothelial cell interaction and smooth muscle cell proliferation and migration [1,2]. In the last years, several studies demonstrated that endothelium-dependent vascular relaxation is reduced in various diseases, such as diabetes, heart failure, coronary heart disease (CHD), hypertension, atherosclerosis and chronic kidney disease (CKD). In these pathologic conditions, NO bioavailability is reduced by oxidation due to excessive production of superoxide anions in the vascular wall, suggesting that antioxidant compounds may have a role in the modulation of endothelial-dependent vasodilatation [3–5].

Epidemiological studies reported that a diet enriched in vegetables and fruits may contribute to reduce the incidence of cancer and cardiovascular disease [6,7]. In particular, a significant reduction in the incidence of CHD was observed in certain areas of France where fat intake is high, leading to the concept of the “French paradox” [8]. The Mediterranean diet, which is characterized by food containing phenols, was also correlated with a reduced incidence and delayed progression of CKD [9,10]. Further studies evidenced a protective effect of dietary intake of phenols contained in red [11,12] or white wine [13–15], and olive oil [16,17]. It has been reported that wine-derived phenols may reduce endothelial dysfunction by enhancing NO availability and/or generation [18–20]. Oral administration of red wine polyphenolic compounds decreased blood pressure in hypertensive rats. This hemodynamic effect was associated with an enhanced endothelium-dependent relaxation and stimulation of inducible NO synthase and cyclooxygenase 2 gene expression within the arterial walls [21]. The cardioprotective activity of red wine has been ascribed mainly to phytocomplex rich in polyphenols, in particular resveratrol (RSV). However, several reports indicated that also white wine may exert cardiovascular protective effects despite the very low content of RSV [22–25]. White wine contains phenols with antioxidant properties such as Caffeic Acid (CAF) and Tyrosol (TYR). In particular, Prasad and co-workers [26] reported that CAF significantly reduced lipid peroxidation and decreased DNA damage in UVB-irradiated lymphocytes. Furthermore, CAF pre-treatment significantly maintains antioxidant status and decreased UVB-induced cytotoxicity[26]. Moreover, Khan et al. [27,28] demonstrated that CAF can reduce oxidative stress and inflammation induced by 12-O-tetradecanoyl-phorbol-13-acetate *in vivo*. However, the effect of these compounds on NO production has not been extensively investigated.

The aim of the present study was to evaluate whether low doses of CAF comparable to those assumed with moderate white wine consumption may exert a protective effect on endothelial injury induced by hypoxia and uremic toxins.

## Materials and Methods

### Human Endothelial Cell Culture

Human umbilical vein-derived endothelial cells (HUVEC) were obtained by ATCC (PCS-100-010—ATCC USA). HUVECs were plated onto flask with EBM medium supplemented with 10% fetal calf serum and different endothelial growth factors as previously reported [29].

HUVECs were cultured for 24 h into an airtight humidified chamber flushed with a gas mixture containing 5% CO_2_, 94% N_2_, and 2% O_2_ at 20 atm, 37°C [30] or with the uremic toxins asymmetric dimethylarginine (ADMA 10 μg/ml from Sigma Aldrich, St. Louis, MO), p-cresyl sulfate (1 μg/ml from Alsachim, Illkirch Graffenstaden, France) and indoxyl sulfate (10 μg/ml from Sigma Aldrich, St. Louis, MO).

### Analysis of NO generation by endothelial cells stimulated with L-Arginine and Acetylcholine

NO generation by HUVECs was detected in chemiluminescence, as previously described [31–32] with some modification. Briefly, HUVECs were cultured in luminometer cuvettes coated with gelatin in appropriate medium and incubated overnight (o.n.) with TYR 100 nM and 1 μM, CAF 100 nM and 1 μM. L-Arginine 1 mM and L-NAME 1 mM were used as positive and negative controls, respectively. At the end of each experiment, culture medium was removed and substituted with HEPES-buffered Krebs medium containing 0.5% BSA, luminol 100 μM (Sigma Aldrich) and hydrogen peroxide (H_2_O_2_) 100 nM; then the cuvettes were inserted into a Luminometer (Berthold, Germany). Baseline chemiluminescence was recorded for 60 seconds and expressed as mean value. Then the cells were stimulated with Ach 100 μM and chemiluminescence was recorded continuously for 180 seconds. Results were expressed as relative light unit (RLU).

### Quantification of NO generation by the fluorescent probe DAF-2 DA

The cell-permeable NO reactive dye, diaminofluorescein 2 diacetate (DAF-2 DA, Alexis Italia/Biochem, Vinci, Italy) was used to examine baseline production of NO in HUVECs plated on gelatin-coated glass-plastic dishes, as previously reported [33].

Cells were washed three times with PBS and were then loaded with DAF-2 DA and/or the phenols at the concentration reported above. Immediately before use, DAF-2 DA stock solution (5 mM) was diluted to a final concentration of 5 μM in PBS-BSA 0.25% (working solution). As control of the eNOS inhibitory activity, HUVECs were incubated with L-NAME for 30 minutes and then with CAF and TYR for 30 minutes.

HUVECs were incubated for 60 min at 37°C within 1 mL/well of the working solution containing L-Arginine (L-Arg) or the phenols and then transferred to a Diaphot inverted microscope (Nikon, Melville, NY) with 20× fluorescent objective in an attached, hermetically sealed Plexiglas NP-2 incubator (Nikon) at 37°C. The microscope was equipped with a video camera connected (Leica, Deerfield, IL) to an IBM-compatible computer. The exposure time was 1.76 sec. Sequential exposures of the same microscopic field were acquired. Image analysis was performed with MicroImage analysis system (Cast Imaging, Venice, Italy). L-NAME 1mM was used as negative control. Quantification of fluorescence intensity was performed by FACS analysis after DAF-2 DA incubation as described below in more details. Experiments were conducted in triplicate.

### FACS Analysis

For FACS analysis, HUVECs were subjected to fixation and permeabilization with 1xPBS containing-1% paraformaldehyde and 0.1% Triton-X, detached with EDTA and stained for 1 h at 4°C with primary antibodies directed to human Akt, P-Akt, eNOS or P-eNOS (Santa Cruz Biotech, Santa Cruz, CA) or with an irrelevant control antibody. After extensive washing, cells were incubated with appropriate Alexa Fluor—conjugated secondary antibodies for 45 min at 4°C. All incubation periods were performed using a medium containing 0.25% BSA and 0.0016% sodium azide. At the end of staining, cells were newly washed, fixed in, and subjected to FACS analysis (Becton Dickinson, Mountain View, CA). Results are expressed as ratio between P-Akt/Akt and P-eNOS/eNOS percentage of positivity.

### ROS production: Lucigenin-enhanced chemiluminescence

Superoxide (O_2˙_^-^) production was revealed by chemiluminescence in presence of lucigenin, as previously described with some modifications [34]. Briefly, HUVECs were cultured in luminometer cuvettes coated with gelatin in appropriate medium, and incubated overnight with RSV 100 nM and 1 μM, TYR 100 nM and 1 μM, CAF 100 nM and 1 μM. At the end of each experiment, culture medium was removed and substituted with HEPES-buffered Krebs medium containing 0.5% BSA and lucigenin 250 μM (Sigma Aldrich). HUVECs were stimulated with the calcium ionophore A23187 (Sigma Aldrich), and the chemiluminescence generated was continuously monitored using a Berthold Luminometer (Germany) for 5 minutes. Results were expressed as mean relative light unit (RLU).

### Image-iT LIVE Green Reactive Oxygen Species (ROS) Detection Kit

Image-iT LIVE Green Reactive Oxygen Species (ROS) Detection Kit was used to analyze oxidative stress on HUVECs as suggested by manufacturer (Life Technologies). Briefly, 5-(and-6)-carboxy-2',7'-dichlorodihydrofluorescein diacetate (carboxy-H_2_DCFDA) was added to HUVECs in different experimental conditions: after 30 min, cells were fixed with 4% paraformaldehyde and then counterstained with Hoechst and analyzed by Immunofluorescence or FACS analysis.

In selected experiments, the protective effect of CAF on HUVEC cultured under hypoxic condition or in presence of uremic toxins was also evaluated.

### Cytotoxicity assay—XTT assay

HUVECs were cultured on 24-well plates (Falcon Labware, Oxnard, CA) at a concentration of 5 × 10^4^ cells/well and incubated for 24h with increasing doses of CAF (Sigma Aldrich) in normoxic, hypoxic culture condition or in presence of uremic toxins. Cells were then incubated with a medium without phenol red additioned with 250 μg/ml XTT (Sigma Aldrich). The absorption values at 450 nm were measured in an automated spectrophotometer at different time points. All experiments were performed in triplicate.

### Detection of apoptosis—TUNEL assay

HUVECs were incubated for 24h with increasing concentrations of CAF (Sigma Aldrich) in normoxia, hypoxia or uremic toxins and then subjected to terminal deoxynucleotidyltransferasemediated dUTP nick end labelling (TUNEL) assay (ApopTag, Oncor, Gaithersburg, MD) that identifies DNA fragmentation, a typical feature of apoptotic cells. Green-stained apoptotic cells were counted in 10 different microscopic fields at ×100 magnification for each experimental point. All experiments were performed in triplicate.

### PBMC Adhesion to HUVEC monolayers

PBMC adhesion to HUVEC monolayers was performed as previously described [35]. PBMC were collected from healthy volunteers using samples obtained by the local blood bank (Banca del Sangue, Azienda Ospedaliera Universitaria Città della Salute e della Scienza di Torino) and discarded from clinical use. A written informed consent was obtained from all subjects in accordance with blood bank guidelines. PBMCs were isolated from healthy volunteers by density gradient and labelled with 10 μm Vybrant cell tracer (Life Technologies, Carlsbad, CA). PBMCs (50 × 10^6^/ml) were added to HUVEC monolayer previously incubated with increasing concentrations of CAF in normoxic, hypoxic condition or in presence of uremic toxins for 12h. After extensive washing to remove detached PBMCs, green-stained cells adherent to HUVEC monolayers were counted in 10 different microscopic fields at ×100 magnification for each experimental point. All experiments were performed in triplicate.

### *In vitro* angiogenesis on Matrigel

The *in vitro* property of HUVECs to form capillary-like structures was studied culturing HUVECs (5 × 10^4^ cells/well) on growth factor—reduced Matrigel (Becton Dickinson. St. Jose, CA) diluted 1:1 in ice with cold DMEM (Sigma, St.Louis, MO—USA) [36]. After extensive washing, different concentrations of CAF were added to HUVECs cultured in normoxia, hypoxia or in presence of uremic toxins. Cells were observed under a Nikon-inverted microscope (Nikon, Kanagawa, Japan). Image analysis was performed with the MicroImage analysis system (Casti Imaging, Venice, Italy)[31].

### Gene array analysis

Human GEarray kit for the study of angiogenesis markers (SuperArray Inc., Bethesda, MD) was used to characterize the gene expression profile of HUVECs cultured under hypoxic condition in presence or absence of 1 μM CAF for 24h. RNA extraction and retro-transcription of total RNA were performed using a commercial kit as suggested by manufacturer (SuperArray). Microarray data were deposited on European Bioinformatic Institute (EBI) website (www.ebi.ac.uk/microarray-as/ae/; experiments archive code: E-MEXP-3908).

### Experimental model of kidney ischemia-reperfusion injury in C57BL-6 mice

C57BL-6 mice were anesthetized by using an induction chamber with isoflurane and by intraperitoneal administration of ketamine (100 mg/kg). Right renal artery and vein were occluded by using a non-traumatic vascular clamp for 30 min. Animals were divided in the following groups: 1) Sham-operated; 2) Ischemia-reperfusion injury (right renal pedicle clamp); 3) Ischemia-reperfusion injury + Caffeic acid (right renal pedicle clamp + 1μM/ml CAF). Six animals from each group were sacrificed 24 hr after renal pedicle clamp. For renal histology, 5-mm-thick paraffin kidney sections were stained with hematoxylin/eosin (Merck, Darmstadt, Germany). Luminal hyaline casts and cell loss (denudation of tubular basement membrane) were assessed in 30 non-consecutive fields using a x40 objective (high-power field: HPF). TdT-mediated dUTP nick end labelling assay (Chemicon International, Temecula, CA) for the detection of apoptotic cells was performed according to manufacturer’s instructions. Granulocyte infiltration was evaluated by staining with an anti-granulocyte antibody (Serotec, Oxford, UK) and immunoperoxidase staining was performed by using an anti-rat HRP (Pierce, Rockford, IL).

### Statistical analysis

All data of different experimental procedures are expressed as average±1SD. Analysis of variance (ANOVA) and Student-Newman-Keuls analysis were used as statistical test where appropriated. For all experiments performed by FACS, Kolmogorov Smirnov statistical analysis was performed. For all analysis, a p value <0.05 was considered statistical significant.

## Results

### Quantification of NO release by chemiluminescence

**Fig 1:**
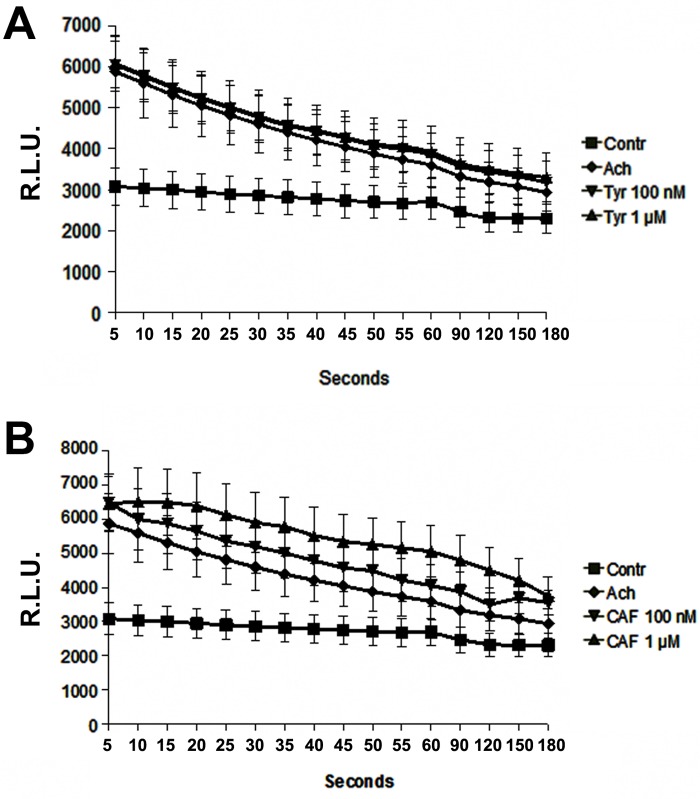
Ach induced NO production by HUVECs. Ach stimulation increased NO release, revealed by chemiluminescence. TYR 1μM and 100nM did not affect Ach-induced NO release (Fig. 1A); CAF enhanced Ach-induced NO release in a dose-dependent manner (Fig. 1B) (p<0.05 CAF vs. controls or vs. Ach at all time points considered). Results are expressed as average±1SD of 6 different experiments.

**Fig 2:**
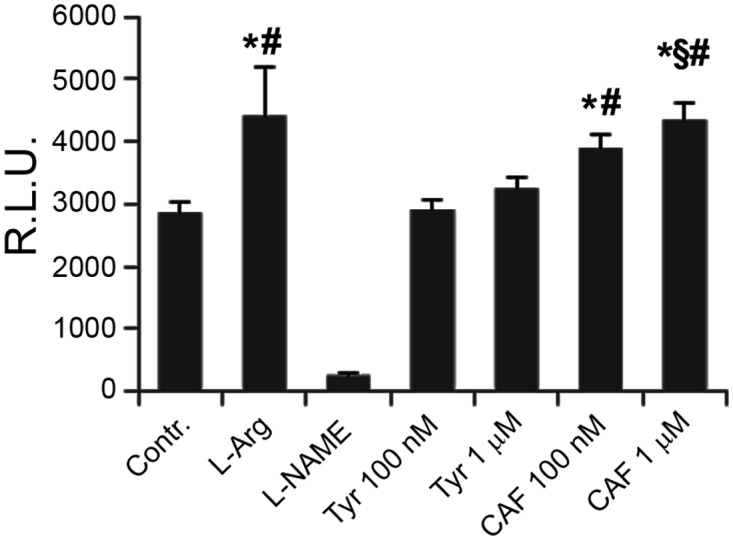
NO-generated chemiluminescence by HUVECs incubated overnight with the phenols. L-Arginine 1 mM and L-NAME 1 mM were used as positive and negative controls, respectively. CAF increased NO production in a dose-dependent manner (*p<0.05 vs controls and L-NAME; § p<0.05 vs CAF 100 nM; # p<0.01 vs controls). Results are expressed as average±1SD of 6 different experiments.

Ach stimulation increased NO production by HUVECs in respect to baseline. Chemiluminescence decreased at every time point recorded even though at the end of observation (180 sec) remained higher than in unstimulated cells (Fig. 1A-B). However, TYR and CAF induced different responses in HUVECs: 100nM and 1μM TYR did not affect Ach-induced NO release (Fig. 1A). By contrast, CAF enhanced Ach-induced NO release in a dose-dependent manner (Fig. 1B). CAF-induced NO production remained at plateau for 25 sec., with a subsequent decrease. We then evaluated in HUVECs the effect of different phenols on NO production independent by Ach stimulation (Fig. 2). L-Arg and L-NAME were used as positive and negative controls, respectively. NO-generated chemiluminescence confirmed the lack of effect of TYR, whereas CAF increased NO production in a dose-dependent manner (Fig. 2).

### Quantification of NO release by the fluorescent probe DAF-2 DA

**Fig 3:**
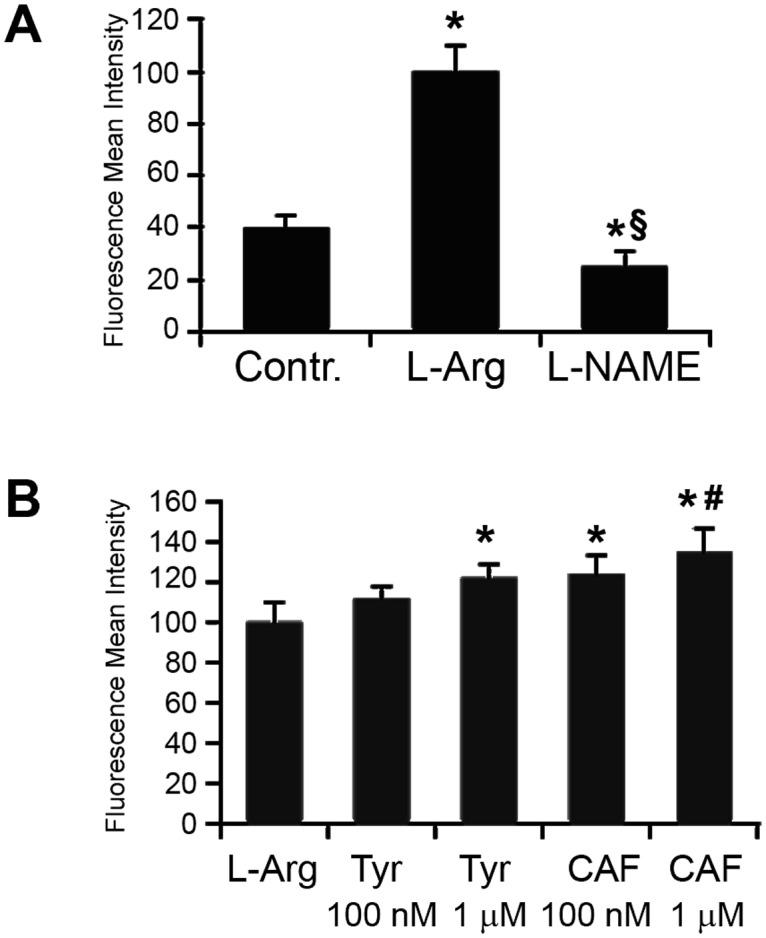
DAF-2 DA detected fluorescence. A) L-Arg stimulated fluorescence emission which peaked at 30 min (*p<0.01 vs controls). L-NAME was utilized as negative control (§p<0.05 vs L-Arg). B) CAF 100 nM and CAF 1 μM enhanced NO dependent fluorescence (*p<0.01 vs controls). CAF 1 μM induced NO release was significantly (#p<0.05) higher in respect to CAF 100 nM. Results are expressed as average±1SD of 6 different experiments.

NO baseline production by HUVECs was also evaluated by quantitative fluorescence emission generated by DAF-2 DA oxidation. L-Arg but not L-NAME stimulated fluorescence emission that peaked at 30 min (Fig. 3A). The analysis of fluorescence intensity showed a significant increase in DAF-2 DA oxidation in respect to L-Arg after incubation with CAF 100nM or 1μM (Fig. 3B) in a dose dependent manner. TYR did not affect NO baseline production.

### Evaluation of eNOS expression and phosphorylation

**Fig 4:**
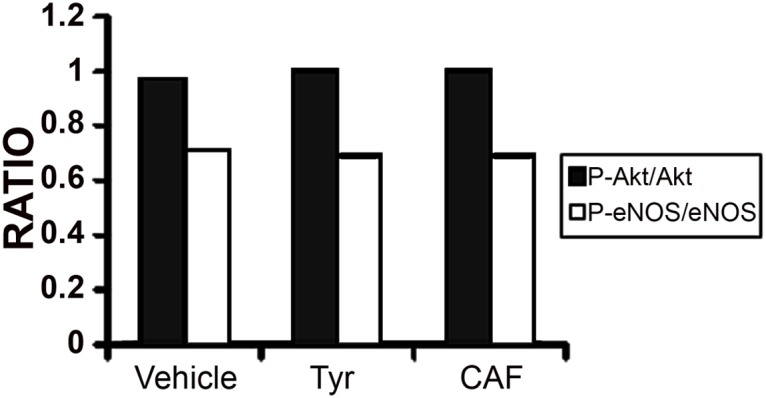
Evaluation of eNOS activation by FACS in HUVECs. Histograms show the ratio between P-eNOS/eNOS and P-Akt/Akt in HUVECs incubated with vehicle alone or with TYR 1μM and CAF 1μM. Results are expressed as ratios of P-eNOS/eNOS and P-Akt/Akt derived from a single FACS experiment. Three different experiments were performed with similar results.

FACS analysis of HUVECs incubated overnight with TYR and CAF did not show any differences in eNOS and Akt expression and phosphorylation when compared to controls stimulated with vehicle alone (Fig. 4).

### ROS production

**Fig 5:**
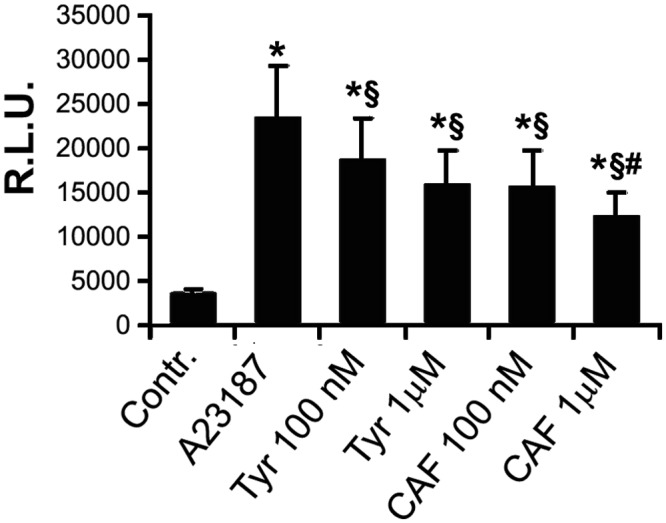
Superoxide production revealed by lucigenin enhanced chemiluminescence in HUVECs incubated with the different phenols and stimulated with the calcium ionofore A23187. CAF and TYR significantly inhibited A23187-induced ROS production (*p<0.001 vs controls, § p<0.01 vs A23187). Results are expressed as average±1SD of 6 different experiments.

The production of ROS was evaluated by enhancement of lucigenin chemiluminescence. HUVECs incubated with A23187 significantly increased ROS production compared to cells challenged with vehicle alone. CAF and TYR significantly inhibited A23187-induced ROS production, suggesting an antioxidant activity (Fig. 5).

### Quantification of NO release and ROS expression in HUVECs cultured in hypoxia or in presence of uremic toxins

In selected experiments, the protective effect of CAF was evaluated on HUVECs cultured under hypoxic condition or in presence of uremic toxins.

**Fig 6:**
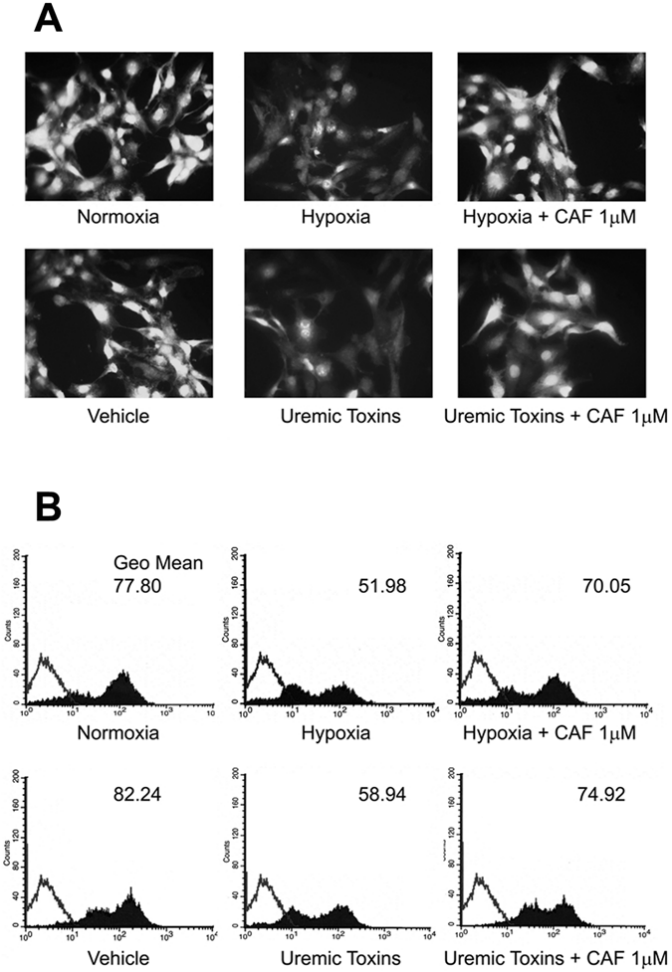
CAF increased NO production by HUVECs cultured in hypoxia or with uremic toxins. Representative fluorescence micrographs (A) and FACS analysis (B) of DAF-2DA probe on HUVECs cultured in hypoxia or with uremic toxins in presence or absence of CAF 1 μM. For immunofluorescence studies, magnification was x400; for FACS analysis, Kolmogorov-Smirnov statistical analysis was performed. For all assays, 5 independent experiments were performed with similar results.

Hypoxia or the uremic toxins ADMA, p-cresyl sulfate and indoxyl sulfate reduced NO release from HUVECs as shown by immunofluorescence studies (Fig. 6A) and FACS analysis (Fig. 6B) using the DAF-2 DA probe. By contrast, CAF 1μM restored NO release from HUVECs cultured in hypoxia or with uremic toxins (Fig. 6A-B).

**Fig 7:**
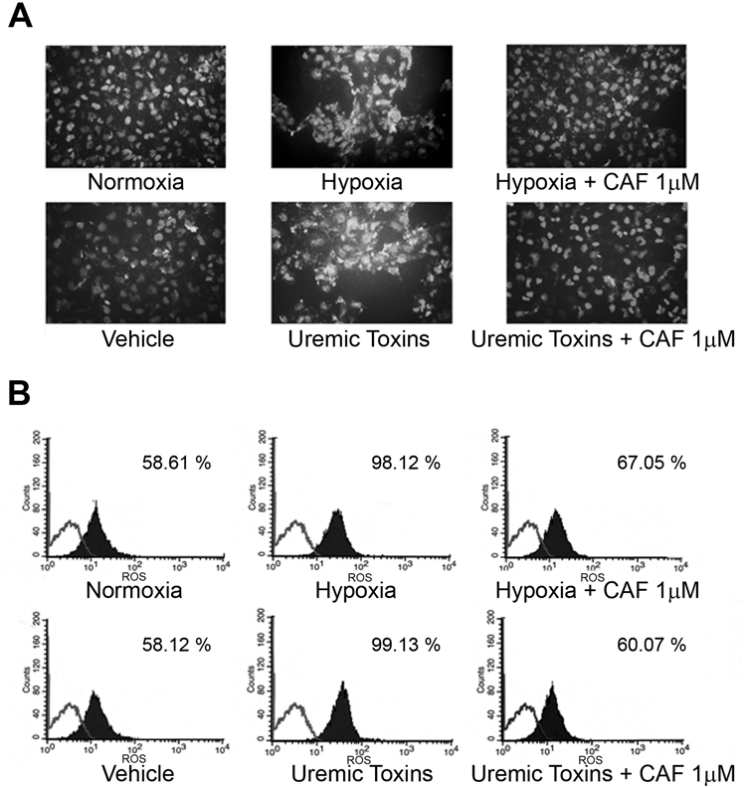
CAF decreased ROS expression in HUVECs cultured in hypoxia or with uremic toxins. Representative fluorescence micrographs (A) and FACS analysis (B) of the Image-iT LIVE Green Reactive Oxygen Species (ROS) Detection Kit on HUVECs cultured in hypoxia or with uremic toxins in presence or absence of CAF 1 μM. For immunofluorescence studies magnification was x200; for FACS analysis, Kolmogorov-Smirnov statistical analysis was performed. For all assays, 5 independent experiments were performed with similar results.

In addition, hypoxia as well as uremic toxins increased ROS production in HUVECs as shown by immunofluorescence studies (Fig. 7A) and FACS analysis (Fig. 7B) using the Image-iT LIVE Green Reactive Oxygen Species (ROS) Detection Kit. CAF 1μM significantly decreased hypoxia- and uremic toxin-induced ROS production by HUVECs (Fig. 7A-B).

### CAF induced proliferation and resistance to apoptosis, reduced leukocyte adhesion and stimulated angiogenesis of HUVECs cultured under hypoxia or in presence of uremic toxins

**Fig 8:**
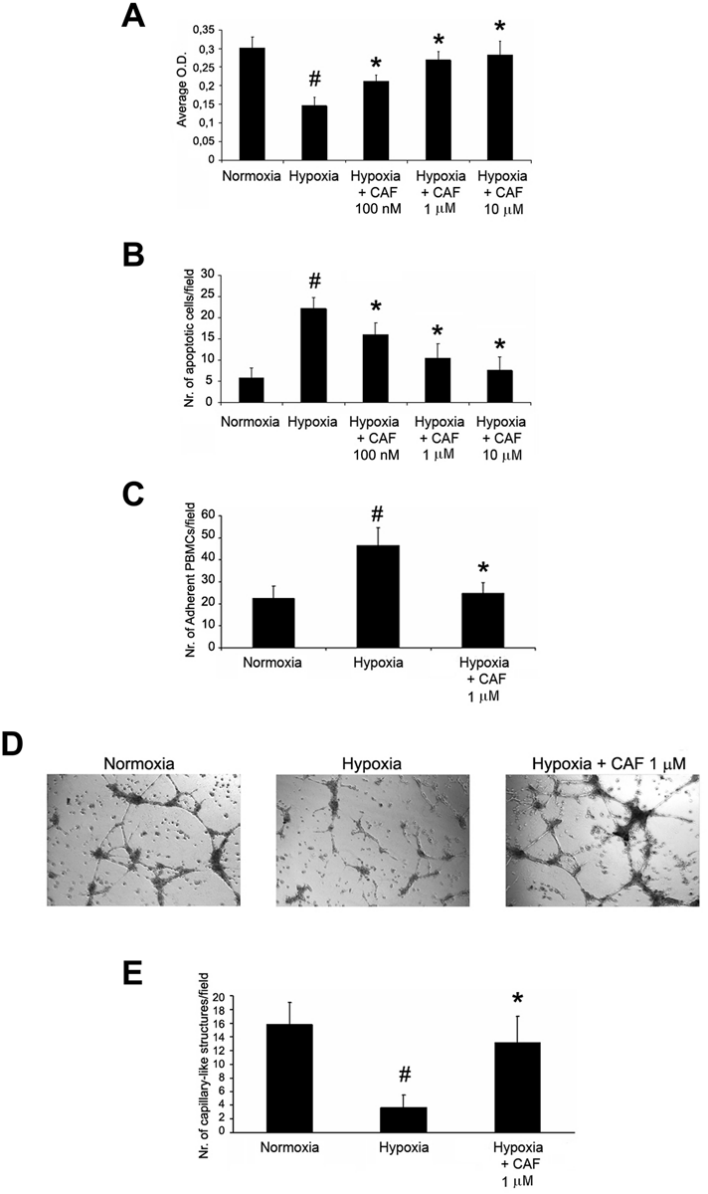
CAF induced proliferation and resistance to apoptosis, decreased PBMC adhesion and triggered *in vitro* angiogenesis of hypoxic HUVECs. Proliferation (XTT-based assay in A), resistance to apoptosis (TUNEL assay in B), PBMC adhesion (C) and *in vitro* angiogenesis on Matrigel coated-plates (representative micrographs in D, count of capillary-like structures in E) of HUVECs cultured in hypoxia in presence or absence of CAF. Hypoxia reduced cell viability, increased apoptosis and PBMC adhesion and abrogated angiogenesis of HUVECs (#p < 0.05 Hypoxia vs. Normoxia). By contrast, increasing doses of CAF (100 nM, 1μM, 10μM) increased viability and resistance to apoptosis (*p < 0.05 Hypoxia + CAF 100 nM, 1μM or 10μM vs. Hypoxia), and a fixed dose of CAF 1μM decreased PBMC adhesion and triggered angiogenesis of hypoxic HUVECs (*p < 0.05 Hypoxia + CAF 1μM vs. Hypoxia). Results are expressed as average±1SD of 3 different experiments.

**Fig 9:**
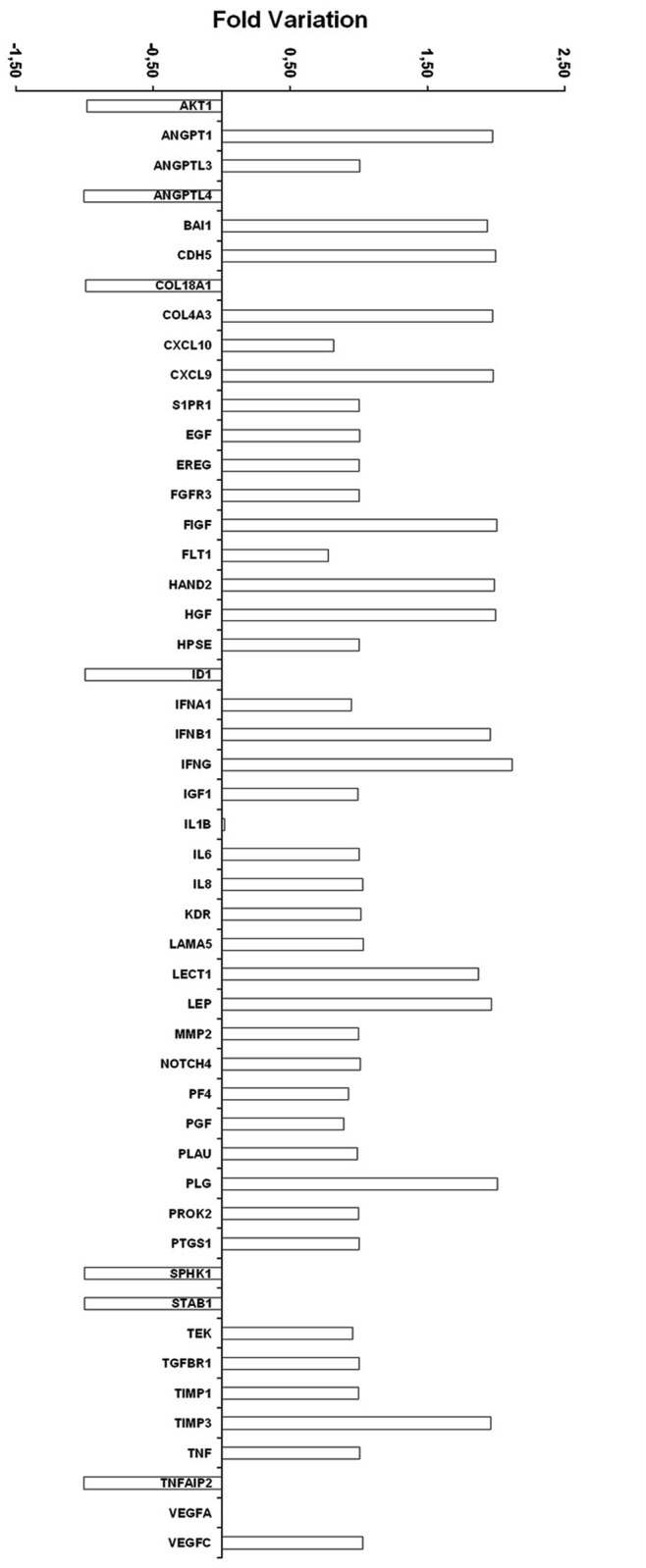
Modulation of gene array profiling of hypoxic HUVECs induced by CAF 1μM (angiogenesis-related genes). The graph shows the fold variation of angiogenesis-related genes between HUVECs stimulated with Hypoxia + CAF 1μM vs. Hypoxia in a single experiment. Samples were normalized for the signals found in housekeeping genes [actin, glyceraldehyde 3 phosphate dehydrogenase (GAPDH)]. Three independent experiments were performed with similar results. Gene table: AKT1, V-akt murine thymoma viral oncogene homolog 1; ANGPT1, Angiopoietin 1; ANGPTL3, Angiopoietin-like 3; ANGPTL4, Angiopoietin-like 4; BAI1, Brain-specific angiogenesis inhibitor 1; CDH5, Cadherin 5, type 2 (vascular endothelium); COL18A1, Collagen, type XVIII, α1; COL4A3, Collagen, type IV, α3 (Goodpasture antigen); CXCL10, Chemokine (C-X-C motif) ligand 10; CXCL9, Chemokine (C-X-C motif) ligand 9; S1PR1, Sphingosine-1-phosphate receptor 1; EGF, Epidermal growth factor; EREG, Epiregulin; FGFR3, fibroblast growth factor receptor 3; FIGF, C-fos induced growth factor (vascular endothelial growth factor D); FLT1, Fms-related tyrosine kinase 1 (vascular endothelial growth factor/vascular permeability factor receptor); HAND2, Heart and neural crest derivatives expressed 2; HGF, Hepatocyte growth factor (hepapoietin A; scatter factor); HPSE, Heparanase; ID1, Inhibitor of DNA binding 1, dominant negative helix-loop-helix protein; IFNA1, Interferon, α1; IFNB1, Interferon, β1, fibroblast; IFNG, Interferon, γ; IGF1, Insulin-like growth factor 1 (somatomedin C); IL1B, Interleukin 1, β; IL6, Interleukin 6 (interferon, β2); IL8, Interleukin 8; KDR, Kinase insert domain receptor (a type III receptor tyrosine kinase); LAMA5, Laminin, α5; LECT1, Leukocyte cell derived chemotaxin 1; LEP, Leptin; MMP2, Matrix metallopeptidase 2 (gelatinase A, 72kDa gelatinase, 72kDa type IV collagenase); NOTCH4, Notch 4; PF4, Platelet factor 4; PGF, Placental growth factor; PLAU, Plasminogen activator, urokinase; PLG, Plasminogen; PROK2, Prokineticin 2; PTGS1, Prostaglandin-endoperoxide synthase 1 (prostaglandin G/H synthase and cyclooxygenase); SPHK1, Sphingosine kinase 1; STAB1, Stabilin 1; TEK, TEK tyrosine kinase, endothelial/Tie-2; TGFBR1 transforming growth factor, β receptor 1; TIMP1, TIMP metallopeptidase inhibitor 1; TIMP3, TIMP metallopeptidase inhibitor 3; TNF, Tumor necrosis factor; TNFAIP2, Tumor necrosis factor, α-induced protein 2; VEGFA, Vascular endothelial growth factor A; VEGFC, Vascular endothelial growth factor C.

**Fig 10:**
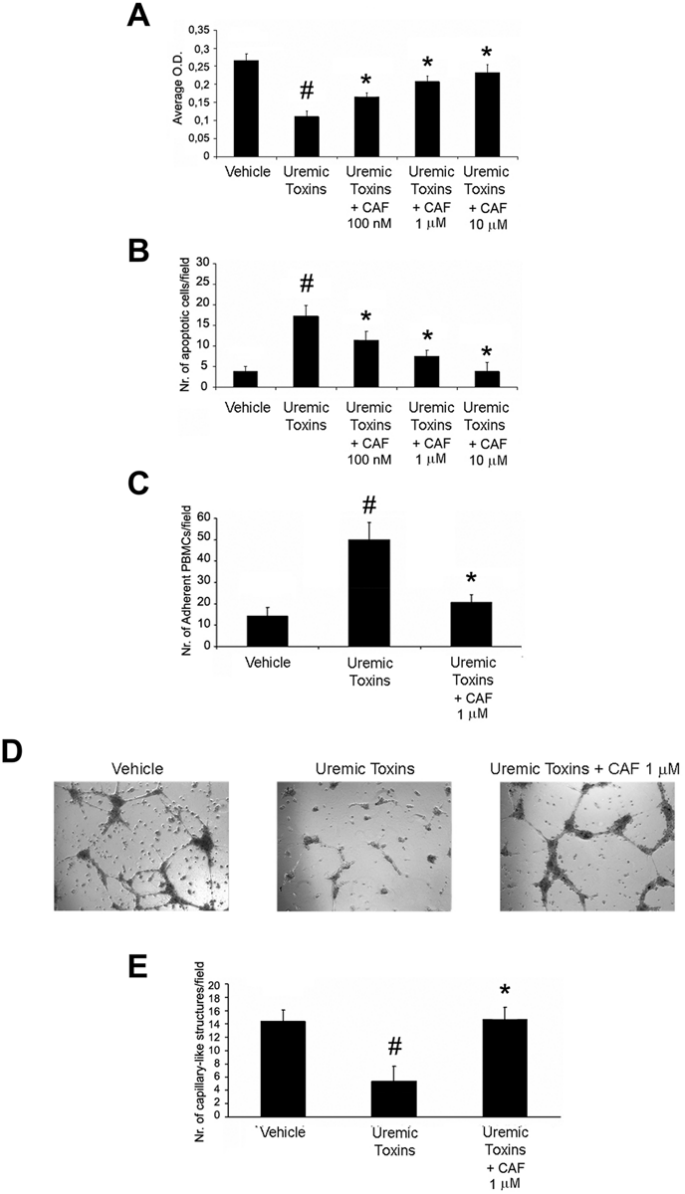
CAF induced proliferation and resistance to apoptosis, decreased PBMC adhesion and triggered *in vitro* angiogenesis of HUVECs cultured with uremic toxins. Proliferation (XTT-based assay in A), resistance to apoptosis (TUNEL assay in B), PBMC adhesion (C) and *in vitro* angiogenesis on Matrigel coated-plates (representative micrographs in D, count of capillary-like structures in E) of HUVECs cultured with the uremic toxins ADMA (10 μg/ml), p-cresyl sulfate (1 μg/ml) and indoxyl sulfate (10 μg/ml) in presence or absence of CAF. Uremic toxins reduced cell viability, increased apoptosis and PBMC adhesion and abrogated angiogenesis of HUVECs (#p < 0.05 Uremic Toxins vs. Vehicle). By contrast, increasing doses of CAF (100 nM, 1μM, 10μM) increased viabilty and resistance to apoptosis (*p < 0.05 Uremic Toxins + CAF 100 nM, 1μM or 10μM vs. Uremic Toxins), and a fixed dose of CAF 1μM decreased PBMC adhesion and triggered angiogenesis of uremic toxin-treated HUVECs (*p < 0.05 Uremic Toxins + CAF 1μM vs. Uremic Toxins). Results are expressed as average±1SD of 3 different experiments.

Increasing doses of CAF (100 nM, 1μM, 10μM) significantly increased proliferation (Fig. 8A), and reduced apoptosis (TUNEL assay in Fig. 8B). In addition, 1μM CAF significantly reduced PBMC adhesion to HUVEC monolayers cultured under hypoxia, suggesting an anti-inflammatory effect (Fig. 8C). CAF also triggered angiogenesis of hypoxic HUVECs as shown in representative micrographs (Fig. 8D) and in count of capillary-like structure formation (Fig. 8E) on Matrigel-coated plates. To further confirm the pro-angiogenic effect of CAF on hypoxic HUVEC, we performed gene array analysis: we found that CAF up-regulated in hypoxic HUVECs the expression of several genes involved in angiogenesis, cell proliferation and resistance to apoptosis (Fig. 9). A similar protective effect of CAF on proliferation (Fig. 10A), resistance to apoptosis (Fig. 10B), PBMC adhesion (Fig. 10C) and triggering of angiogenesis (Fig. 10D-E) was also observed in HUVECs cultured in presence of the uremic toxins ADMA, p-cresyl sulfate and indoxyl sulfate known to induce endothelial injury and apoptosis through the induction of oxidative stress [37–40].

### CAF reduced tubular injury and granulocyte infiltration after kidney ischemia-reperfusion injury

**Fig 11:**
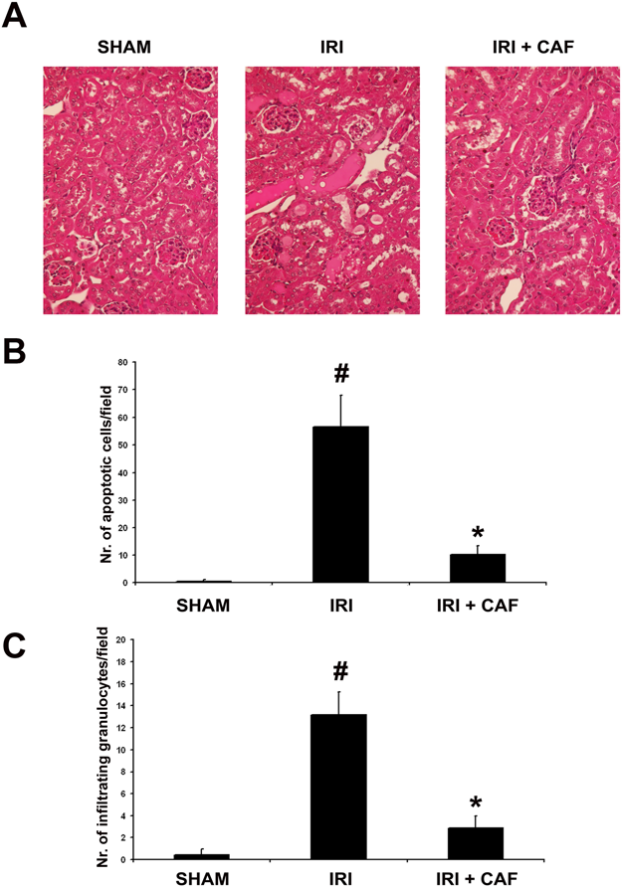
CAF reduced tubular cell injury and granulocyte infiltration in an experimental model of kidney ischemia-reperfusion injury in C57BL-6 mice. (A) Hematoxylin/eosin staining of representative kidney sections from different experimental groups of C57BL-6 mice (n = 6 for each group): sham-operated (SHAM); right kidney ischemia-reperfusion injury (IRI); right kidney ischemia-reperfusion injury + Caffeic acid 1μM/ml (IRI + CAF). Original magnification was x40. Counts of TdT-mediated dUTP nick end labeling (TUNEL)-positive cells (B) and of granulocyte infiltration (C) in the different experimental conditions. A significant increase of apoptotic tubular cells and infiltrating granulocytes was observed in IRI in comparison with sham-treated animals (#p<0.05 IRI vs. SHAM). CAF induced a significant decrease of apoptotic tubular cells and granulocyte infiltration in mice subjected to IRI (*p<0.05 IRI + CAF vs. IRI). In B and C, results are expressed as average±1SD in 30 non-consecutive fields.

In comparison to sham-operated animals, mice subjected to kidney ischemia-reperfusion injury showed histological signs of tubular injury such as formation of hyaline casts, vacuolization, diffuse necrosis and denudation of basal membrane. When mice were treated with 1μM/ml CAF, a significant reduction of tubular injury was observed (Fig. 11A and Table 1). In addition, CAF significantly reduced the number of apoptotic tubular cells (Fig. 11B) and granulocyte infiltration (Fig. 11C) within ischemic kidney.

**Table 1:** Morphologic evaluation of tubular injury in different experimental conditions.

	Casts (n/HPF)	Tubular necrosis (n/HPF)
**Sham**	0	0
**IRI**	10.21 ±3.74	17.54 ±5.09
**IRI + CAF**	3.63 ±1.82*	7.12 ±3.39*

Abbreviations: IRI, ischemia—reperfusion injury; CAF caffeic acid; n/HPF, number/high-power field.

Results are expressed as average±1SD of 30 non-consecutive fields.

* p<0.05 IRI+ CAF vs. IRI.

## Discussion

In the present study, we demonstrated that CAF modulates endothelial NO production in a dose-dependent manner. These results may suggest a potential role of CAF in the inhibition of oxidant stress-associated endothelial dysfunction induced by hypoxia or by incubation with the uremic toxins ADMA, p-cresyl sulfate and indoxyl sulfate. This potential protective effect of CAF was confirmed in an experimental model of kidney ischemia-reperfusion injury in which this compound significantly reduced tubular cell apoptosis and granulocyte infiltration.

We first observed that CAF induced an enhancement of basal and Ach-induced NO production by HUVECs. This effect was not due to an increased expression of eNOS but rather to a scavenger^.^ activity of CAF. Indeed, we did not observe an increased phosphorylation of eNOS in endothelial cells treated with CAF, suggesting that NO production was not directly stimulated by this compound. Furthermore, the experiments performed with the pro-oxidant A23187 confirmed the anti-oxidant and scavenger activity of CAF.

Previous studies demonstrated that CAF has a potential anti-atherogenic activity through the reduction of cell adhesion molecule expression and chemokine production by HUVECs incubated with inflammatory cytokines such as TNF-α [41]. In the present study, we demonstrated that CAF, at doses comparable with those observed after moderate white wine consumption [42–43], induced NO production in endothelial cells [28, 42, 44, 45]. The efficacy of CAF in modulating NO metabolism has also been recently confirmed in an experimental model of cerebral infarction in adult male New Zealand rabbits [46]. These results are in accordance with previous studies showing CAF-induced vasorelaxation in isolated rat aorta [47], whereas other phenols such as TYR were ineffective [48]. TYR is an extensively investigated phenol present in wine and olive oil, main components of the Mediterranean diet. TYR is known to exert anti-oxidant activities in different *in vitro* studies and experimental models [48]. Our results confirmed the anti-oxidant properties of TYR. However, TYR was less effective than CAF in NO production by HUVECs, suggesting that CAF may be one of the key mediators involved in the reduction of endothelial dysfunction observed in subjects adherent to Mediterranean diet [49].

The typical endothelial dysfunction could be ascribed either to eNOS inhibition or to an increased superoxide production [50]. In our experiments, the increase of endothelial NO bioavailability induced by CAF was not due to an enhancement of eNOS expression but rather to an anti-oxidant activity in accordance with previous studies [51]. Basing on these observations, we investigated the protective effect of CAF on oxidant stress-induced endothelial dysfunction in two different *in vitro* models using HUVECs cultured under hypoxia or with known uremic toxins such as ADMA, p-cresyl sulfate and indoxyl sulfate. Indeed, hypoxia as well as uremic toxins plays a crucial role in CKD progression through a pro-inflammatory, pro-apoptotic and anti-angiogenic activity on endothelial cells [52].

We observed that increasing doses of CAF reduced the cytotoxic and pro-apoptotic effect induced by hypoxia on HUVECs. Moreover, CAF significantly reduced the adhesion of leukocytes on hypoxia-stimulated HUVEC monolayers, suggesting a role in the inhibition of the inflammatory endothelial response typical of ischemia-reperfusion injury [53]. Indeed, in the present study, we found that in an experimental model of acute kidney injury (AKI) due to ischemia-reperfusion, CAF significantly reduced the percentage of tubular cell apoptosis as well as the number of infiltrating granulocytes. These results suggest a dual protective role of CAF on hypoxia-associated tubular injury and inflammation.

Ischemic-induced tissue hypoxia is a condition shared by different pathologic conditions associated with endothelial dysfunction including AKI, CHD and CKD [53]. Indeed, it has been shown that alteration of oxygen pathways and NO play a key role in the pathogenesis of acute kidney injury (AKI) and in the cellular mechanisms of progression toward CKD [54]. In particular, chronic hypoxia in the renal tubulo-interstitial compartment is responsible for the hemodynamic changes and for the altered oxygen metabolism of resident kidney cells that lead to the typical histological findings of CKD such as peritubular capillary rarefaction, tubular atrophy and interstitial fibrosis [55]. Hypoxia causes endothelial apoptosis and promotes epithelial-to-mesenchymal transition through the increased expression of extracellular matrix proteins and oxidative genes [56]. Moreover, it has been shown that uremia induces an alteration of oxygen consumption in kidney resident cells leading to the deterioration of renal function through oxidative stress [55–56]. Our results suggest a protective effect of CAF in the course of events leading to oxidative stress-induced endothelial injury and CKD progression in presence of hypoxia. In addition, it has been shown that several uremic toxins, biologically active uremic retention molecules known to accelerate renal dysfunction, are able to deregulate oxygen metabolism, thus worsening the hypoxic damage and the activation of the renin-angiotensin-aldosterone system [57]. Among uremic toxins, guanidino compounds are known to induce endothelial production of ROS, cytotoxicity and calcification [58]. Asymmetric dimethylarginine (ADMA) is a key mediator of uremia-associated endothelial dysfunction and triggering of atherosclerosis. ADMA is an endogenous L-arginine analogue acting as inhibitor of NO synthase whose levels are significantly increased in end stage renal disease patients. In addition, elevated ADMA levels are associated with an enhancement of CKD progression and with an increased incidence of cardiovascular and cerebrovascular diseases due to oxidative stress damage [59]. Other protein bound uremic toxins such as p-cresyl sulfate and indoxyl sulfate are increased in uremic patients and are known to induce an intense production of ROS and senescence of endothelial cells [60]. Moreover, indoxyl sulfate inhibits NO production and facilitate leukocyte endothelial interaction through the up-regulation of E-selectin, ICAM-1 and MCP-1 on endothelial cell surface [61]. A similar effect of indoxyl sulfate as well as of p-cresyl sulfate has also been observed on kidney tubular epithelial cells through ROS production, activation of NF-kB and p53 and production of TGF-beta able to enhance the epithelial-to-mesenchymal transition [62]. As described for hypoxia-induced damage, we found that CAF, at low doses present in the white wine, protected endothelial cells from apoptosis and functional alterations induced by ADMA, p-cresyl sulfate and indoxyl sulfate at levels found in the uremic milieu. In particular, CAF increased cell proliferation, resistance to apoptosis and *in vitro* angiogenesis that were all reduced after treatment with uremic toxins. In addition, CAF significantly reduced the adhesion of leukocyte to HUVECs incubated with uremic toxins.

CAF-associated endothelial protection was mainly ascribed to the inhibition of ROS expression and to the increase of NO release. However, gene array analysis revealed that CAF was able to modulate the endothelial expression of several genes involved in angiogenesis and inhibition of apoptosis of hypoxic endothelial cells independently by NO production. In particular, CAF induced the endothelial expression of different growth factors and angiogenic molecules such as angiopoietin-1, EGF, FGF, HGF, IGF-1, IL-6, VEGF, MMP-2 and growth factor receptors including FGF-R, Flt1 and KDR.

In conclusion, the results of the present study suggest that CAF-induced NO bioavaibility and inhibition of ROS production may explain, at least in part, the potential endothelial protection associated with moderate white wine assumption typical of Mediterranean diet. The CAF-induced inhibition of the inflammatory and apoptotic injury and the triggering of angiogenesis of HUVECs may be responsible for a cardio-protective and reno-protective effect of this phenol due to the limitation of oxidant stress-induced microvascular derangement in presence of hypoxia and/or uremic toxins.

## Supporting Information

S1 Data Results of experiments. (XLS)
